# Elevated expression of syntenin in breast cancer is correlated with lymph node metastasis and poor patient survival

**DOI:** 10.1186/bcr3442

**Published:** 2013-06-20

**Authors:** Yu Yang, Qi Hong, Pengcheng Shi, Zhebin Liu, Jianmin Luo, Zhiming Shao

**Affiliations:** 1 Breast Cancer Institute, Cancer Hospital, Fudan University, Shanghai 200032, P.R. China; 2 Institutes of Biomedical Sciences, Fudan University, Shanghai 200032, P.R. China

**Keywords:** breast cancer, syntenin, metastasis, ERK1/2, patient survival

## Abstract

**Introduction:**

Syntenin is a scaffolding-PDZ domain-containing protein. Although it is reported that syntenin is associated with melanoma growth and metastasis, the possible role of syntenin in breast cancer has not been well elucidated. The present study investigated the expression and function of syntenin in breast cancer.

**Methods:**

Real-time polymerase chain reaction (PCR) and Western blots were used to determine the mRNA and protein expression of syntenin. With a combination of overexpression and RNA interference, the effect of syntenin on migration, invasion, and ERK1/2 activation was examined in breast cancer cell lines. The effect of syntenin *in vivo *was assessed with an orthotropic xenograft tumor model in BALB/c nu/nu mice. In addition, the expression level of syntenin in clinical breast cancer tissues was evaluated with immunohistochemistry. The Kaplan-Meier survival curve was used to evaluate patient survival, and the Cox proportional hazards model was used for multivariate analysis.

**Results:**

Our study showed that syntenin expression was upregulated in high-metastasis breast cancer cell lines and breast cancer tissues. Overexpression of syntenin in breast cancer cells promoted cell migration and invasion *in vitro*. Moreover, overexpression of syntenin promoted breast tumor growth and lung metastasis *in vivo*. We further showed that activation of integrin β1 and ERK1/2 was required for syntenin-mediated migration and invasion of breast cancer cells. The correlation between syntenin expression and tumor size (*P *= 0.011), lymph node status (*P *= 0.001), and recurrence (*P *= 0.002) was statistically significant. More important, syntenin expression in primary tumors was significantly related to patients' overall survival (OS; *P *= 0.023) and disease-free survival (DFS; *P *= 0.001). Its status was an independent prognostic factor of OS (*P *= 0.049) and DFS (*P *= 0.002) in our cohort of patients.

**Conclusions:**

These results suggest that syntenin plays a significant role in breast cancer progression, and it warrants further investigation as a candidate molecular marker of breast cancer metastasis and a potential therapeutic target.

## Introduction

Breast cancer is the most common malignancy in women, and metastasis is the major cause of mortality [1]. Although many molecules have been implicated in breast cancer occurrence and progression, the detailed mechanism is still not completely understood. Syntenin is a scaffolding-PDZ domain-containing protein involved in multiple biologic functions, including syndecan binding and recycling, receptor clustering, protein trafficking, signal transduction, and regulation of the transcription factor Sox4 proteasomal degradation [2-7]. The PDZ domains of syntenin bind to multiple peptide motifs with low-to-medium affinity [8,9], are essential for assembly and organization of diverse cell-signaling processes occurring at the plasma membrane [6,10]. More recently, many reports described interactions between syntenin and a large number of proteins, including class B ephrins, pro-transforming growth factor-α, phosphotyrosine phosphatase-D, neurofaschin, neurexin, schwannomin (also known as merlin), IL-5 receptor, various glutamate receptor subtypes, the syndecan family of heparan sulfate proteoglycans, and ubiquitin [6,11], indirectly implicating its role in a variety of cellular processes.

Extensive studies have shown that syntenin is upregulated in several cancer cells and tissues and may regulate tumor cell invasion and metastasis [3,12-14]. Boukerche and colleagues [3,15] found that syntenin could increase FAK, JNK and p38 MAP kinase activity, as well as activation of nuclear factor-kappaB (NF-κB), all of which play an important role in syntenin-mediated anchorage-independent growth and motility in melanoma. In contrast to the observations in melanoma, overexpression of syntenin resulted in increased phosphorylation of AKT S473 and extracellular signal-regulated kinase ERK1/2 in HEK 293T cells [13]. Moreover, inhibition of p38 or JNK MAPK did not have any effect on syntenin-induced invasion in HEK 293T cells [13]. This discrepancy might be explained by cell type-specific action of syntenin. The diversity of syntenin-interaction partners also suggests that syntenin may have flexible cell-type-specific roles.

Although the function of syntenin in melanoma has been partially revealed, little is known about the expression of syntenin and what the molecular mechanisms are in breast cancer. In the present study, we found an elevated expression of syntenin in high-metastasis breast cancer cell lines and breast cancer tissues. Moreover, forced syntenin overexpression promoted cell migration and cell invasion, suggesting a possible role of syntenin in metastatic spreading of breast cancer cells. Consistent with *in vitro *results, we observed that syntenin overexpression promoted breast tumor growth and lung metastasis *in vivo*. We further showed that syntenin-mediated migration and invasion of breast cancer cells required the activation of integrin β1 and ERK1/2. The correlation between syntenin expression and tumor size, lymph node status, and recurrence was statistically significant. More important, immunohistochemistry staining showed that the expression level of syntenin in primary tumors was significantly related to patient survival in our cohort of patients.

## Materials and methods

### Reagents

Antibodies specific for syntenin (sc-100336) and GAPDH were purchased from Santa Cruz Biotechnology (Santa Cruz, CA, USA). Antibodies against ERK1/2, phospho-ERK1/2, JNK, phospho-JNK, p38, and phospho-p38 were obtained from Cell Signaling Technology (Beverly, MA, USA). Secondary antibodies, HRP-conjugated Goat anti-Mouse IgG, Goat anti-Rabbit IgG, and anti-integrin β1 (MAB2079Z for Western blot and MAB1959 for functional blocking) were obtained from Millipore (Billerica, MA, USA). MAB2079Z is specific for active conformation of human integrin β1.

Cell-culture media and all supplements were purchased from Invitrogen (Basel, Switzerland). All reagents for gel electrophoresis were obtained from Bio-Rad (Reinach, Switzerland). U0126 was purchased from Sigma Chemical Co. (St. Louis, MO, USA). Matrigel was purchased from BD Biosciences (San Diego, CA, USA).

### Cell culture

Human breast cancer cell lines MCF7, ZR-75-1, ZR-75-30, MDA-MB-468, MDA-MB-435, and MDA-MB-231 were purchased from the American Type Culture Collection (Manassas, VA, USA). The highly metastatic MDA-MB-231HM cell line was established by subclone-selection procedure in our institute, and its establishment was described previously [16,17]. The MDA-MB-231HM and MDA-MB-435 cell lines have high metastatic potential to the lung. MDA-MB-231, MDA-MB-231HM, MDA-MB-468, and MDA-MB-435 cells were grown in Leibovitz L-15 medium; ZR-75-1 and ZR-75-30 cells were maintained in RPMI 1640 medium; and MCF-7 cells were maintained in DMEM medium. All media were supplemented with 10% fetal bovine serum (FBS), penicillin (100 units/ml), and streptomycin (0.1 mg/ml). All cells were incubated at 37°C in a humidified 5% CO_2 _atmosphere.

### Real-time quantitative PCR

Total RNA was extracted by using TRIzol reagent (Invitrogen, Carlsbad, CA, USA), and reverse transcription was performed with a PrimeScript RT reagent Kit (Takara Biotechnology, Dalian, China). Gene-specific primers for human syntenin and GAPDH were generated by Sangon Biotech (Shanghai) Co., Ltd. The primer sequences were as follows: human syntenin (forward primer, 5'- TTCTGCTCCTATCCCTCACG-3' and reverse primer, 5'- CCAGTTACAGGAGCCACCAT -3'), human GAPDH (forward primer, 5'- TGCCAAATATGATGACATCAAGAA-3' and reverse primer, 5'-GGAGTGGGTGTCGCTGTTG-3'). The iCycler and iQ real-time PCR (Bio-Rad, Hercules, CA, USA) and SYBR-Green PCR Master Mix (Toyobo, Osaka, Japan) were used for amplification and detection. The qPCR condi¬tions were as follows: 95°C for 5 minutes; and 95°C for 30 seconds, 60°C for 30 seconds, and 72°C for 30 seconds for 39 additional cycles. Data were recorded and analyzed by using the real-time PCR analysis software Bio-Rad iQ5. All experiments were repeated thrice, and the mRNA levels of each gene were normalized to that of GAPDH as an internal control.

### Western blot

Cell lysates were prepared in RIPA buffer with protease inhibitors (complete mini protease inhibitor; Roche) and phosphatase inhibitor cocktail (Sigma-Aldrich). The lysates were then clarified by centrifugation at 12,000 *g *for 10 minutes at 4°C. Protein concentration was measured with the BCA assay (Thermo Scientific). Equivalent amounts of protein were subjected to SDS-PAGE analysis. Immunoblotting was performed by using standard methods. Quantification of the protein was conducted by the Image Analyzer LAS-3000 (FujiFilm Life Science, Tokyo, Japan). The image was analyzed with densitometry. Each sample was normalized to GAPDH.

### Cloning and stable overexpression of syntenin

The coding sequence of syntenin (897 bp) was cloned from normal human cDNA. We cloned syntenin into the pCDH-CMV-MCS-EF1-PURO expression vector (System Biosciences). Constructs were confirmed by sequencing. Production of the lentiviral particles was carried out according to the manufacturer's protocol. MDA-MB-231 cells were infected with the lentiviral particles according to the manufacturer's protocol. The empty vector was packaged as a negative control. Stable transfectants were selected and cultured in medium containing 3 µg/ml puromycin.

### Stable RNA interference against syntenin

We designed short-hairpin RNA molecules targeted against sites beginning at nucleotides 262 and 622 of syntenin. The following oligonucleotides were synthesized (Sangon, Shanghai):

1. position 262, 5-ccggCCTATCCCTCACGATGG-AAATctcgagATTTCCATCGTGAGGGATAGGtttttg-3 and 5-aattcaaaaaCCTATC-CCTCACGATGGAAATctcgagATTTCCATCGTGAGGGATAGG-3;

2. position 622, 5-ccggGTACTTCAGATCAATGGTGAActcgagTTCACCATTGATCTGAAG-TACtttttg-3 and 5-aattcaaaaaGTACTTCAGATCAATGGTGAActcgagTTCACC-ATTGATCTGAAGTAC-3 (lower-case letters represent linkers).

Oligonucleotides were annealed and inserted into digested pLKO.1-TRC Cloning Vector plasmid. Production of the lentiviral particles was carried out according to the manufacturer's protocol. MDA-MB-231HM cells were infected with lentivirus particles containing the shRNA, and stable transfectants were selected and cultured in medium containing 3 µg/ml puromycin. The pLKO.1-scramble shRNA plasmid was packaged as a negative control. The digested pLKO.1-TRC Cloning Vector plasmid, packaging plasmid, pCMV-dR8.s2 dvpr, and envelope pCMV-VSVG were purchased from Addgen (Cambridge, MA, USA).

### Scratch assay

Cells were seeded in 60-mm culture dishes at 1 × 10^5 ^cells/dish. A scratch through the central axis of the plate was gently made by using a pipette tip. Migration of the cells into the scratch was observed at time points of 24 and 48 hours.

### Migration and invasion assay

Migration assay and invasion assay were conducted with a 24-well cell-culture chamber by using inserts with 8-μm pores (Corning Costar). The breast cancer cell lines were suspended in L-15 medium without FBS at 1× 10^5^/ml (migration) or 1× 10^6^/ml (invasion), and 0.1 ml of the suspension was added in triplicate to the inserts. L-15 medium with 10% FBS was added into the lower chamber. After a 4-hour (migration) or 36-hour (invasion) incubation at 37°C, the cells on the lower surface of the membrane were stained with Giemsa stain and counted with microscopy in five different fields (200×).

### Animal experiments

The animals used in this study were 4- to 6-week-old athymic female BALB/c nu/nu mice, which were provided by Shanghai Institute of Materia Medica, Chinese Academy of Science. For mammary-fat-pad tumor assays, cells were harvested by trypsinization, washed twice in PBS, and counted. Cells were then resuspended (10^7 ^cells/ml) in a 1:1 ratio of PBS and gelatinous protein (Matrigel). Mice were anesthetized, a small incision was made to reveal the mammary gland, and 10^6 ^cells were injected directly into the mammary fat pad. The incision was closed with wound clips, and primary tumor outgrowth was monitored weekly by taking measurements of the tumor length (L) and width (W). The animals were killed and autopsies performed 6 weeks after tumor inoculation. The tumor volumes were calculated by using the following formula: volume = 0.52 × width^2 ^× length. Metastasis formation was assessed with microscopic observation of the lung tissue slices. All animal experiments were carried out with approval from the Shanghai Medical Experimental Animal Care Commission, Fudan University.

### Immunohistochemistry

Immunohistochemistry (IHC) staining of syntenin was performed on formalin-fixed, paraffin-embedded sections from 239 patient tumor samples with known clinical history (Table 1). Informed-consent forms were obtained from all the patients. This study was approved in advance by the Institutional Review Board of the Cancer Hospital, Fudan University. Tumor sections were incubated in a 1:100 dilution of anti-syntenin antibody and a 1:50 dilution of anti-CD31 antibody. Negative control consisted of phosphate-buffered saline instead of primary antibody. Antibody sensitivity and specificity were verified by IHC staining on formalin-fixed, paraffin-embedded tumors formed by syntenin-overexpression and syntenin-knockdown cells. Tumors formed by 231-SYN were used as positive controls in IHC staining of human breast cancer tissues.

**Table 1:** Clinicopathologic characteristics of the patients

	Syntenin expression		
	
	Negative (*n *= 151)	Positive (*n *= 88)	*P *value
Mean age (years)	53.6	52.35	0.357
Tumor size			0.011^a^
T1	21	9	
T2	121	63	
T3	9	16	
Grade			0.518
I	1	2	
II	111	64	
III	33	17	
Lymph node status			0.001 ^a^
Negative	138	67	
Positive	13	21	
ER			0.891
Negative	57	34	
Positive	94	54	
PR			0.134
Negative	55	41	
Positive	96	47	
Her-2			0.414
Negative	62	37	
Positive	75	44	
Not available	3		
Recurrence			0.002^a^
No	118	52	
Yes	33	36	

^a^*P *< 0.05.

Negative control showed no brown staining, and positive control showed specific staining with little or no background staining, indicating a successful IHC. Multiple microscopic fields (200×) of each tumor section were analyzed. The results were read semiquantitatively by two independent investigators, who were blinded to the patient outcomes. Discrepancies between the two observers were reviewed jointly to reach consensus.

The intensity of syntenin immunohistochemistry was estimated and graded in a four-step scale as not stained (−), slightly stained (+), moderately stained (++), or intensely stained (+++). The results were classified into two categories: positive (intensely stained or moderately stained, 20% or more than 20% positive cells) and negative (slightly stained or not stained, less than 20% of positive cells).

### Statistical analysis

ANOVA and Student *t *tests were used to determine the statistical significance of differences between experimental groups. The Kaplan-Meier method was used to analyze breast cancer patient cumulative survival rate. Cox proportional hazards model was used to analyze the independent prognostic factor for breast cancer. Values of *P*<0.05 were considered statistically significant. Statistical analysis and graphs were created with GraphPad Prism (Graph-Pad Software, San Diego, CA, USA).

## Results

### Expression of syntenin in high-metastasis breast cancer cell lines and breast cancer tissues

**Figure 1:**
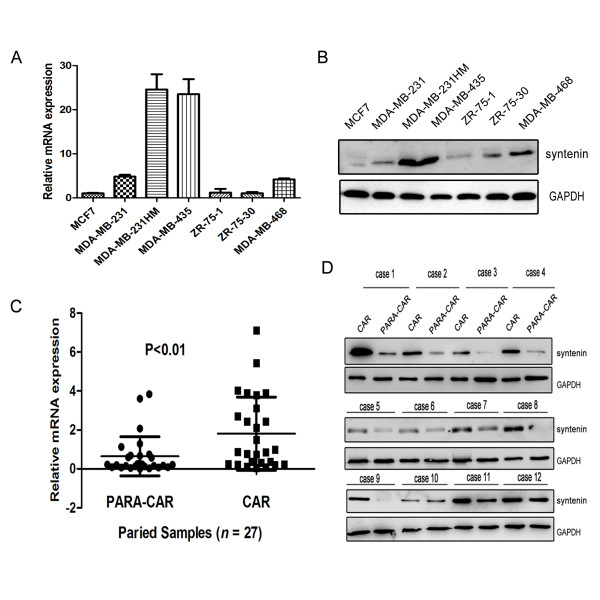
**Syntenin expression is upregulated in high-metastasis breast cancer cell lines and breast cancer tissues**. **(A) **Real-time PCR analysis of syntenin mRNA expression in breast cancer cell lines. **(B) **Western blots show the protein expression level of syntenin in breast cancer cell lines. **(C) **Comparison of syntenin mRNA expression in 27 paired carcinomas (CARs) and paracarcinomas (PARA-CARs). Results represent mean ± SD (*P *< 0. 01). **(D) **Representative Western blots results show that carcinoma significantly elevated syntenin expression compared with paracarcinoma. The remaining samples results are showed in Additional file 1, Figure 1S. Relative mRNA levels were normalized to GAPDH mRNA. Protein loading was monitored with immunoblot by using GAPDH antibodies.

We performed real-time PCR and Western blots to investigate the mRNA and protein expression levels of syntenin in human breast cancer cell lines. As shown in Figure 1A and 1B, MDA-MB-231HM cells had higher syntenin expression than their parent cells and low-invasive cells, such as MCF-7, ZR-75-1, ZR-75-30, and MDA-MB-468. Meanwhile, the high-metastatic-potential cell line MDA-MB-435 also had higher expression of syntenin than did low-invasive cell lines. These findings suggest a potential correlation between syntenin expression and the metastatic ability of breast cancer cell lines.

Next, we confirmed that syntenin mRNA and protein were also notably higher in breast carcinoma compared with that in corresponding paracarcinoma tissues in 27 paired breast cancer samples (Figure 1C, D, and Additional file 1, Figure S1).

### Syntenin overexpression promotes migration and invasion of breast cancer cells *in vitro*

To quantify the direct contribution of syntenin in breast cancer, we generated a 231-SYN cell line, which stably overexpressed syntenin in MDA-MB-231 cells with a lentivirus-mediated system. A control cell line carrying empty vector was also obtained and termed 231-VEC. The overexpression was verified with real-time PCR (Additional file 1, Figure S2A) and Western blot (Additional file 1, Figure S2B). Meanwhile, we generated MDA-MB-231HM cells stably expressing syntenin shRNA. Two independent sites were targeted, beginning at nucleotide 262 and 622, to generate 231HM-262 cells and 231HM-622 cells, respectively. The negative control expressing scrambled shRNA was named 231HM-VEC. The efficiency of knockdown was determined with real-time PCR (Additional file 1, Figure S2C) and Western blot (Additional file 1, Figure S2D).

**Figure 2:**
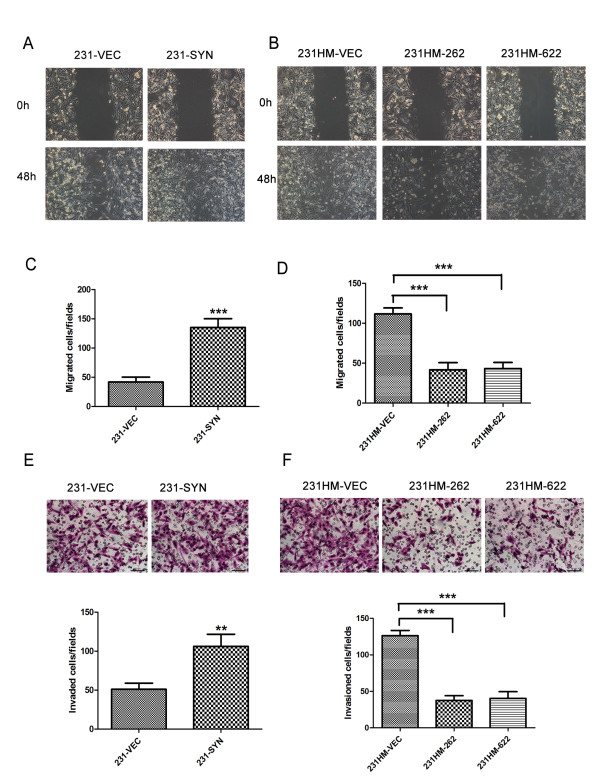
**Effect of syntenin on breast cancer cell migration and invasion *in vitro***. **(A, B) **Cell migration assessed by scratch assay. Representative area of cells at 48 hours after scratch assay is shown. **(C, D) **Migration assay. **(E, F) **Invasion assay. (C, E) Overexpression of syntenin promoted breast cancer cell migration (C) and invasion (E). (D, F) The migrated or invaded cells were significantly reduced after syntenin knockdown in MDA-MB-231HM cells. Cells were plated on noncoated or gelatinous protein (Matrigel)-coated filters for migration or invasion assay, respectively. Cells that migrated or invaded through the pores in the filter were fixed, stained, and counted in five random fields visualized with microscopy (200×). Data represent mean ± SD of three independent experiments performed in triplicate. **P < 0.01; ***P < 0.001.

Scratch assay and Transwell migration assay were performed to test whether syntenin expression influences the migratory behavior of cells. The 231-SYN cells exhibited greatly enhanced migration ability compared with either the MDA-MB-231 or 231-VEC cells (*P *< 0.001, Figure 2A and 2C). Consistent results were observed in MDA-MB-231HM cells (*P *< 0.001, Figure 2B and 2D). We further investigated the role of syntenin in cell invasion by using an invasion assay coated with Matrigel, and found that syntenin overexpression also promotes breast cancer cell invasion (*P *< 0.01; Figure 2E and 2F). These results indicated that syntenin was involved in breast cancer cell migration and invasion.

### Syntenin overexpression promotes tumor growth and lung metastasis *in vivo*

**Figure 3:**
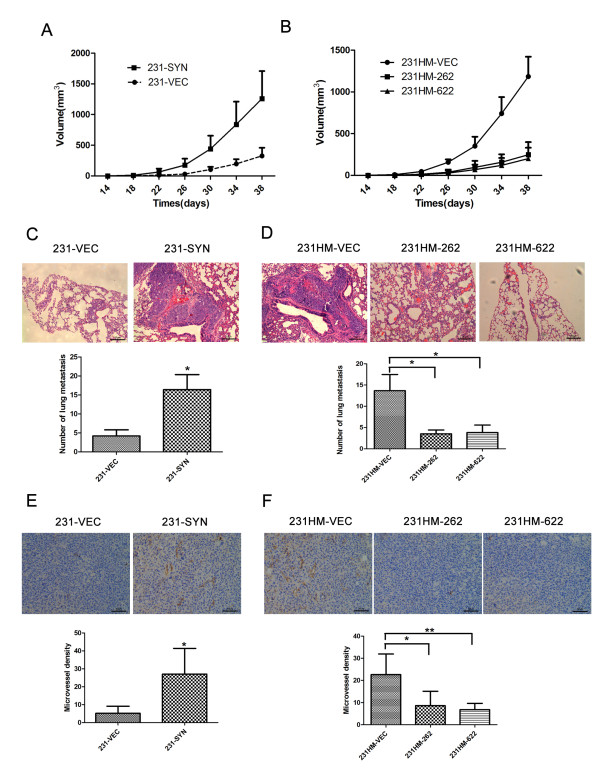
**Syntenin overexpression promotes breast tumor growth and lung metastasis *in vivo***. **(A) **Overexpression of syntenin promoted breast tumor growth in nude mice (*P *< 0.01). **(B) **Volumes of tumors formed by syntenin-knockdown cells were significantly smaller than control. Bars, SEM. Five mice in each group were used in this study. **(C, D) **Hematoxylin-eosin (HE) was used to examine the number of lung metastasis (200×). Quantification of average metastasis number of per lung (*n *= 5). **(E, F) **Immunohistochemistry examination of CD31 for microvessels in the final week of xenografts (200×). Quantification of average microvessel density per xenografts (*n *= 5). *P < 0.05.

To determine whether syntenin could promote tumor growth and accelerate metastasis *in vivo*, we used orthotopic xenograft tumor models in the nude mice. As shown in Figure 3A, 231-SYN cells formed larger tumors than control 231-VEC cells (*P *< 0.01). Consistent results were obtained by mammary fat pad implantation of 231HM-VEC, 231HM-262, and 231HM-622 (*P *< 0.01; Figure 3B). These results showed that syntenin overexpression could promote tumor growth *in vivo*.

The number of lung metastatic nodules was counted to determine whether the overexpression of syntenin in breast cancer cells could enhance lung metastasis *in vivo*. Hematoxylin-eosin (HE) stain was used to detect the lung metastasis. As shown in Figure 3C, 231-SYN cells led to more metastatic nodules than did the vector control cells (*P *< 0.05). Similarly, mice injected with the 231HM-262, 231HM-622 cells exhibited fewer pulmonary metastatic nodules than did those injected with control cells (Figure 3D). These data demonstrate that syntenin overexpression could promote breast cancer cells metastasis to the lung. All the primary tumors were evaluated by pathologic examination.

We next examined whether syntenin expression affects angiogenesis. The results showed that microvessel density was significantly increased in tumors formed by syntenin-overexpressing cells and decreased in tumors formed by syntenin-knockdown cells (Figure 3E and 3F). These data suggest that syntenin could enhance tumor growth by promoting blood vessel formation.

### Elevated syntenin expression induces integrin β1 and ERK1/2 MAPK activation

**Figure 4:**
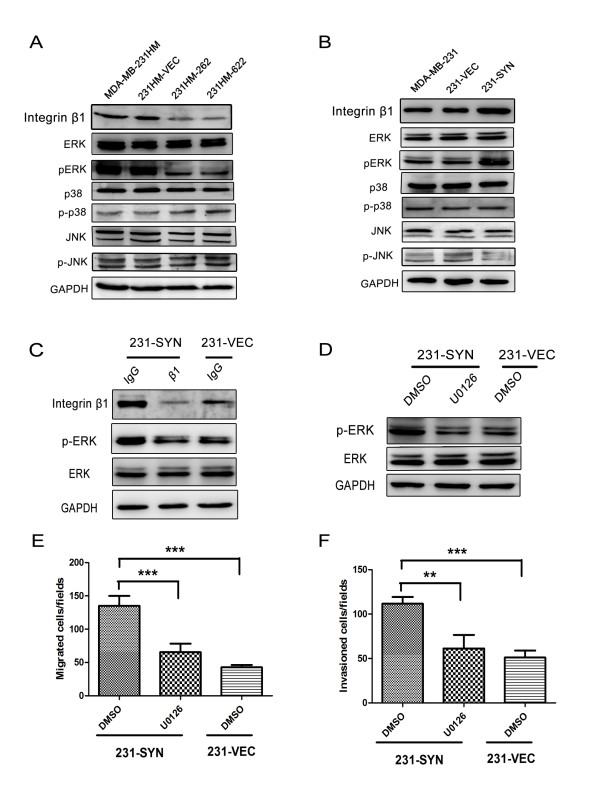
**Activation of integrin β1 and ERK1/2 is essential for syntenin-induced migration and invasion**. **(A) **Silencing of syntenin in MDA-MB-231HM cells inhibited active integrin β1 expression and phosphorylation of ERK1/2, but had no effects on JNK and p38. **(B) **Overexpression of syntenin increased active integrin β1 expression and ERK1/2 phosphorylation in 231-SYN cells. The activation of ERK1/2 was analyzed with Western blot by using phosphor-specific antibodies. **(C) **Integrin β1 functional blocking antibody blocked both active integrin β1 expression and ERK1/2 phosphorylation. Cells were treated with integrin β1 or nonspecific IgG for 1 hour before protein extraction. **(D) **ERK1/2 inhibitor U0126 blocked activation of ERK1/2. Cells were pretreated with dimethylsulfoxide (DMSO) or 20 µ*M *U0126 (U0126) for 2 hours before protein extraction. **(E, F) **U0126 effectively reduced the migration and invasion of breast cancer cells. GAPDH was used as loading control. Data are expressed as means of triplicate samples from three independent experiments; bars, SD. **P < 0.01; ***P < 0.001.

To investigate the molecular mechanism by which syntenin induces cell migration and invasion in breast cancer cells, we analyzed the effect of syntenin on MAPK activation. As shown in Figure 4A, knockdown of syntenin in MDA-MB-231HM cells significantly reduced the phosphorylation levels of ERK1/2. In contrast, overexpression of syntenin in MDA-MB-231 cells increased ERK1/2 phosphorylation (Figure 4B). However, either overexpression or knockdown of syntenin had no effect on JNK and p38 MAPK activation (Figure 4A and 4B). These results suggest that syntenin overexpression induced ERK1/2 MAPK activation in breast cancer cells.

We further investigated how syntenin activates ERK1/2 at the plasma membrane. Activation of the ERK1/2 cascade is mostly initiated at membrane receptors, such as receptor Tyr kinases (RTKs), G protein-coupled receptors (GPCRs), integrins, ion channels, and so on. Previous study showed that syntenin positively regulates the ILK adaptor function for the assembly of integrin β1/IPP signaling complexes, which activate integrin signaling pathways, including AKT, ERK1/2, and Rac1, in breast cancer cells [18]. Additionally, a study reported that syntenin could assemble multimeric integrin β1 signaling complexes to activate FAK in response to FN in MDA-MB-231 cells [19]. As shown in Figure 4A and 4B, we found that knockdown of syntenin in MDA-MB-231HM cells significantly reduced active integrin β1 expression, whereas overexpression of syntenin in MDA-MB-231 increased active integrin β1 expression.

We next determined whether syntenin-induced alterations of integrin β1 expression are responsible for ERK1/2 phosphorylation. We found that blocking integrin β1 signaling by using a function-blocking antibody diminished both active integrin β1 expression and syntenin-induced ERK1/2 phosphorylation (Figure 4C). These results suggest that integrin β1 expression was involved in syntenin-induced ERK activation.

### Inhibition of MEK/ERK pathway blocks syntenin overexpression-induced cell migration and invasion

Further to investigate the role of ERK1/2 activation in migration and invasion induced by syntenin overexpression, 231-SYN cells were treated with ERK1/2 inhibitors (U0126) for 2 hours. 231-VEC cells were treated with DMSO as a control. Remarkably, cells treated with U0126 significantly blocked ERK1/2 activation (Figure 4D) and the induction of migration and invasion by syntenin overexpression (Figure 4E and 4F). The data reveal an essential role for ERK1/2 MAPK signaling in syntenin-induced migration and invasion in 231-SYN cells.

### Syntenin expression is correlated with breast cancer patient survival

**Figure 5:**
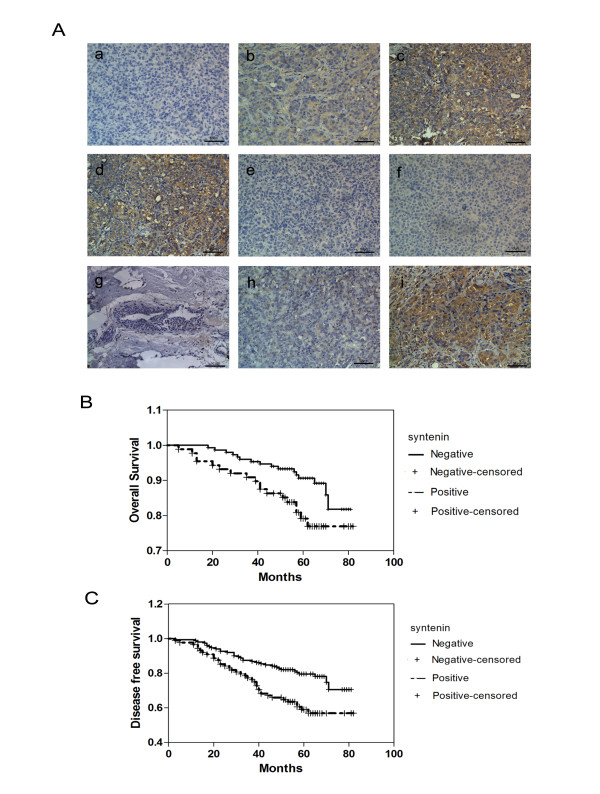
**Elevated expression of syntenin correlates with poor patient survival**. **(A) **Representative images of syntenin immunohistochemistry (IHC) staining. a: Negative control consisted of phosphate-buffered saline instead of syntenin antibody. b-f: IHC staining of paraffin-embedded tumors formed by 231-VEC (b), 231-SYN (c), 231HM-VEC (d), 231HM-262 (e), and 231HM-622 (f) cells. g-i: Representative images of paracarcinoma (g) and carcinoma (h: negative, i: positive). **(B, C) **Kaplan-Meier survival analyses of breast cancer patients. (B) OS (*P *= 0.023). (C) DFS (*P *= 0.001). *P *values were calculated by using the log-rank test.

To validate the antibody used in immunohistochemistry (IHC) staining, we performed IHC staining of paraffin-embedded tumors formed by 231-VEC, 231-SYN, 231HM-VEC, 231HM-262, and 231HM-622 cells. The results showed that tumors formed by 231-SYN and 231HM-VEC cells were intensely stained, whereas tumors formed by 231-VEC, 231HM-262, and 231HM-622 cells were weakly stained or not stained (Figure 5A). These results are consistent with the real-time PCR and Western blot results, and reveal that the antibody used in IHC staining was sensitive and specific.

To determine whether syntenin expression in primary tumor tissues is associated with clinical characteristics (Table 1), we performed immunohistochemistry staining on samples from 239 patients. Paracarcinoma did not show syntenin immunoreactivity (Figure 5A). Representative images of syntenin-positive and -negative staining are presented in Figure 5A. As shown in Table 1, 88 (36.8%) samples were syntenin positive in our cohort of patients. The correlation between syntenin expression and tumor size (*P *= 0.011), lymph node status (*P *= 0.001), and recurrence (*P *= 0.002) was statistically significant, supporting the conclusion that syntenin is involved in breast cancer progression and metastasis. However, syntenin expression had no relation with ages, grade, human epidermal growth factor receptor-2 (Her-2), estrogen receptor (ER), and progesterone receptor (PR) status (*P *> 0.05).

To assess whether syntenin is a prognostic indicator for breast cancer patients, we plotted Kaplan-Meier survival curves and conducted log-rank tests for overall survival (OS) and disease-free survival (DFS). The results indicated that the expression level of syntenin was correlated with OS (*P *= 0.023; Figure 5B) and DFS (*P *= 0.001; Figure 5C). Further to investigate whether syntenin is an independent prognostic factor, we performed multivariate analysis with the Cox proportional hazards model. The results showed that syntenin status was an independent prognostic factor for OS (hazard ratio (HR): 2.031 (95% CI, 1.002 to 4.117); *P *= 0.049, Table 2] and DFS [HR: 2.281(95% CI 1.355 to 3.841), *P *= 0.002, Table 3] in our cohort of patients.

**Table 2:** Multivariate analysis of overall survival by Cox proportional hazards models

Patients	*P*	Hazard ratio (95% confidence interval)
Tumor size T1 versus T2 versus T3	0.076	--
Grade, I versus II versus III	0.769	--
Lymph node status, negative versus positive	0.381	--
Syntenin, negative versus positive	0.049	2.031 (1.002-4.117)

**Table 3:** Multivariate analysis of disease-free survival by Cox proportional hazards models

Patients	*P*	Hazard ratio (95% confidence interval)
Tumor size T1 versus T2 versus T3	0.77	--
Grade, I versus II versus III	0.326	--
Lymph node status, negative versus positive	1.356	--
Syntenin, negative versus positive	0.002	2.281 (1.355-3.841)

## Discussion

In the present study, we provide the first evidence that syntenin expression is upregulated in breast carcinoma, and the elevated expression of syntenin confers a high risk of recurrence. In addition, our results suggest a role of syntenin overexpression in promoting tumor growth and metastatic spreading in breast carcinoma. Recently, observations indicated that syntenin is upregulated in several cancer cells and tissues and may regulate tumor cell invasion and metastasis, including gastric carcinomas [12], melanoma [14,20,21], and brain cancer [22]. Our results were consistent with those previous findings.

Cell migration and invasion are important steps of tumor metastasis. Metastasis is a complex multistep, and multigenic regulated process, in which tumor cells alter its cell-cell adhesion, interact with and break down the surrounding extracellular matrix or the basement membrane, and migrate through the remodeled ECM to reach adjacent organs in response to chemoattractants [13]. In our study, we found that syntenin overexpression could promote breast cell migration and invasion *in vitro*, but the fold changes in migration assay and invasion assay were similar. It suggests that the major role of syntenin maybe in cell migration, not in ECM degradation. Moreover, syntenin overexpression did not alter the expression of matrix metalloproteinases such as MMP-2 and -9, or their proteolytic activities, as assessed by gelatin zymography, which supports that syntenin promotes metastasis of cancer cells mainly through increasing their migratory activity [12]. Syntenin could remodel the actin cytoskeleton, induce the formation of a variety of plasma membrane structures, including goffer, lamellipodia, exosomes, and neurite-like structures in neurons and different types of tumor cells, also indicating that it may play a role in tumor cell migration [23-26]. Orthotropic xenograft tumor models in nude mice further supported that syntenin could accelerate metastasis *in vivo*. These results suggest that syntenin play an important role in tumor lung metastasis.

Although the role of syntenin in regulating cell migration, invasion, and metastasis has been confirmed by multiple studies, several discrepant findings have been observed. Studies in HEK 293T cells revealed the importance of both PDZ domains in conferring syntenin function, whereas in breast cancer cells, only the PDZ2 domain has been shown to be relevant [12,13]. In the context of melanoma, syntenin functions as a positive regulator of melanoma progression and metastasis through interaction with c-Src and promotes the formation of an active FAK/c-Src signaling complex, leading to activation of NF-kappa B and subsequent induction of genes involved in migration and invasion [27]. The activation of p38 and JNK MAPK plays an important role in syntenin-mediated anchorage-independent growth and motility in melanoma [3]. However, Ras, Rho-Rac, PI3K/AKT, and ERK MAPK signaling were shown to mediate syntenin function in HEK293T cells [3,13,15,28]. Inhibition of p38 or JNK MAPK did not have any effect on syntenin-induced invasion in HEK293T cells [13]. In our study, we found that syntenin-mediated migration and invasion of breast cancer cells required the activation of integrin β1and ERK1/2 MAPK. This discrepancy may be explained by the cell type studied and different environmental contexts. The diversity of syntenin-interaction partners also suggests that syntenin may have flexible cell-type-specific roles.

Four dominating MAPK signaling pathways are found in breast disease and function in mammary epithelial cells, including ERK1/2 pathway, JNK pathway, p38 pathway, and ERK5 pathway [29,30]. The ERK1/2 pathway has been shown to promote cell motility and invasion [31], and is implicated in a number of human breast cancer and in many experimental models of breast cancer progression [30]. Because syntenin overexpression increased the phosphorylation level of ERK1/2, we believe that ERK1/2 might modulate the increased migration and invasion of breast cancer cells. Inhibition of ERK1/2 activation with U0126 significantly reduced both expression of phosphorylated ERK1/2 and syntenin-induced migration and invasion. These data revealed an essential role for the ERK1/2 signaling pathway in syntenin-induced migration and invasion in breast cancer cells.

We further investigated how syntenin activates ERK1/2 at the plasma membrane, and found that integrin β1 was involved in syntenin-induced ERK1/2 activation. Taken together, these results suggest that syntenin could activate ERK1/2 through an integrin/RAS/RAF/MEK/ERK signaling pathway, and ERK1/2 activation was essential for syntenin-induced migration and invasion in breast cancer cells.

Although syntenin overexpression had no effect on cell proliferation *in vitro*, it promoted tumor growth *in vivo*. It might be ascribed to angiogenesis regulated by syntenin overexpression. Angiogenesis is a complex process that appears to be important for tumor growth and metastatic potential [32]. A recent study demonstrated that syntenin is a key regulator of angiogenesis and regulates the expression of several proteins responsible for promoting angiogenesis, including insulin growth factor binding protein-2 (IGFBP-2) and interleukin-8 [33]. Moreover, several studies have shown that ERK activation is involved in angiogenesis [34-36]. In our study, we found that syntenin overexpression could induce both ERK activation and angiogenesis in breast cancer. Therefore, it is reasonable for us to believe that the ERK MAPK pathway may be involved in syntenin-induced angiogenesis.

More important, we performed IHC staining on human breast cancer samples. In our 239-case study, we found a significant correlation between syntenin expression level and tumor size, lymph node status, and recurrence. The correlation between syntenin expression and metastasis was consistent with our *in vivo and vitro *experiments. The Kaplan-Meier survival analysis showed that syntenin expression was correlated with OS and DFS. In multivariate analysis with Cox proportional hazards model, we found syntenin status was an independent prognostic factor for OS and DFS in breast cancer patients. These results further supported that syntenin is involved in breast cancer progression and positively correlated with metastasis.

In conclusion, our present data indicate that syntenin plays an important role in breast cancer metastasis and progression both *in vitro *and *in vivo*. The expression level of syntenin is positively correlated with patient survival. These findings suggest that syntenin deserves further investigation as a potential target for novel therapies aiming to block the metastatic process in this tumor.

## Conclusions

Our study shows that syntenin expression is upregulated in high-metastasis breast cancer cell lines and breast cancer tissues. Syntenin overexpression promotes breast cancer cell migration and invasion *in vitro*. Moreover, syntenin overexpression promotes breast tumor growth and lung metastasis *in vivo*. Activation of integrin β1 and ERK1/2 are essential for syntenin-induced migration and invasion *in vitro*. The correlation between syntenin expression and tumor size, lymph node status, and recurrence is statistically significant. The expression level of syntenin in breast cancer tissues was significantly correlated with OS and DFS in our cohort of patients. These results suggest that syntenin plays a significant role in breast cancer progression and is positively correlated with metastasis.

## Abbreviations

DFS: disease-free survival; DMSO: dimethyl sulfoxide; ER: estrogen receptor; HEK 293T: human embryonic kidney cell line 293T; HER 2: human epidermal growth factor receptor 2; MAPK: mitogen-activated protein kinase; OS: overall survival; PR: progestin receptor.

## Competing interests

The authors declare that they have no competing interests.

## Authors' contributions

ZMS and YY conceived, designed, and analyzed the study. YY carried out most of the experiments and wrote the main body of the manuscript. QH generated lentiviral constructs and discussed the data. PCS and ZBL retrieved the patient data from the records. The manuscript was critically revised by PCS. JML helped with IHC statistical analysis. All authors read and approved the manuscript for publication.

## Supplementary Material

Additional file 1 **Figure S1 Comparison of syntenin protein expression in the rest of 15 paired carcinomas (CARs) and paracarcinomas (PARA-CARs)**. Protein loading was normalized to GAPDH. Figure S2 Lentivirus-mediated syntenin expression changes in breast cancer cell lines. (A, B) Syntenin overexpression in MDA-MB-231 cells was detected with real-time PCR and Western blot. **(C, D) **Real-time PCR and Western blot analysis of syntenin knockdown in MDA-MB-231HM cells. Relative mRNA levels were normalized to GAPDH mRNA. Protein loading was normalized to GAPDH. Columns, mean of three independent experiments; bars, SD. Click here for file
