# Supplementary figures and images for: Elevated expression of syntenin in breast cancer is correlated with lymph node metastasis and poor patient survival
Source: Breast Cancer Res. 2013 Jun 20;15(3):R50. doi: 10.1186/bcr3442 (PMC4053163; doi:10.1186/bcr3442)

Figure S1

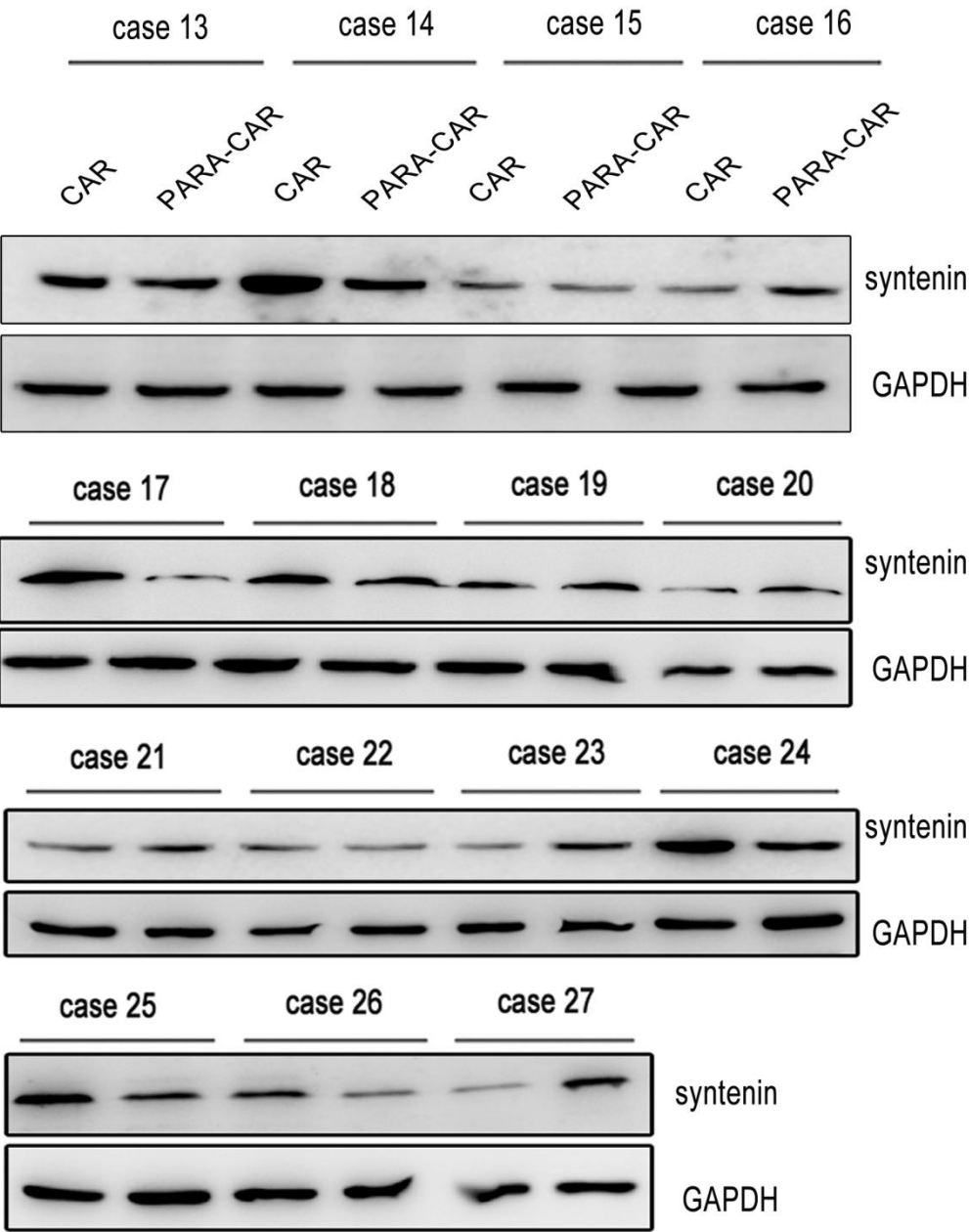

Figure S2

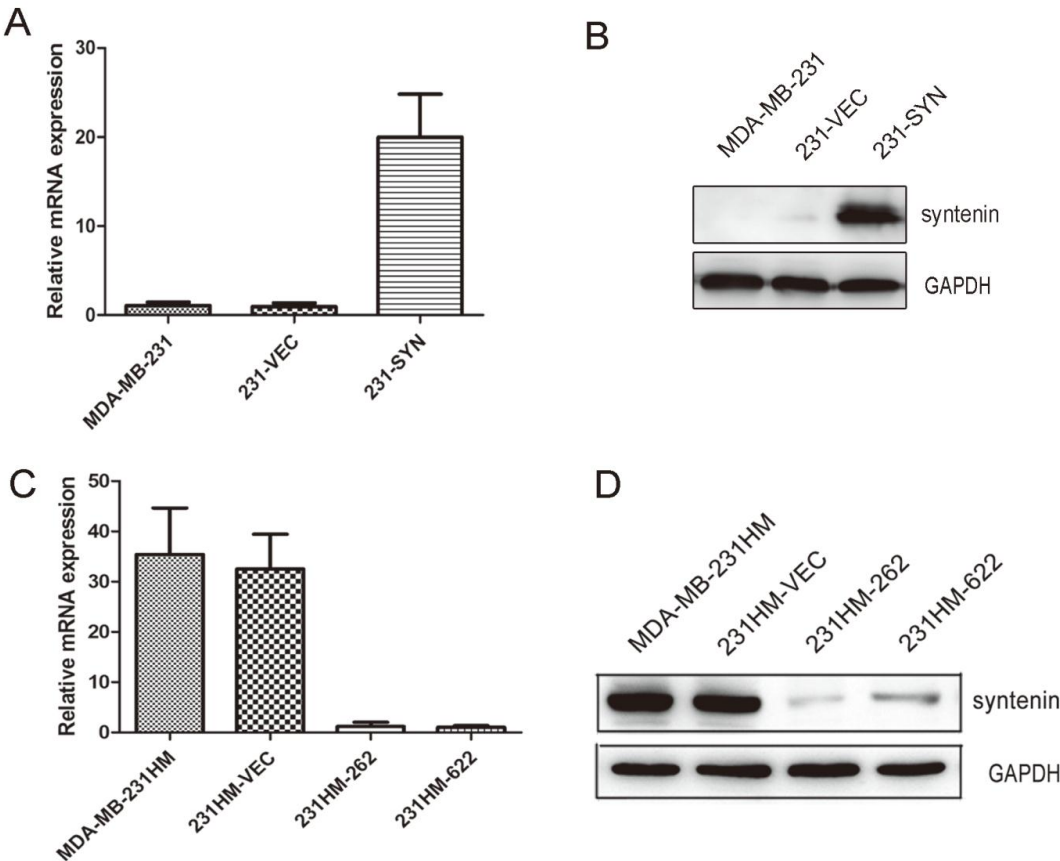

Supplement: Additional file 1 — Figure S1 Comparison of syntenin protein expression in the rest of 15 paired carcinomas (CARs) and paracarcinomas (PARA-CARs). Protein loading was normalized to GAPDH. Figure S2 Lentivirus-mediated syntenin expression changes in breast cancer cell lines. (A, B) Syntenin overexpression in MDA-MB-231 cells was detected with real-time PCR and Western blot. (C, D) Real-time PCR and Western blot analysis of syntenin knockdown in MDA-MB-231HM cells. Relative mRNA levels were normalized to GAPDH mRNA. Protein loading was normalized to GAPDH. Columns, mean of three independent experiments; bars, SD. [file bcr3442-S1.PDF]
